# Tuberculous osteo-arthritis unmasked through unusual elbow swelling: A case report

**DOI:** 10.1016/j.ijscr.2024.110759

**Published:** 2024-12-24

**Authors:** Faten Limaiem, Mohamed Amine Gharbi, Leila Bouhajja, Ramzi Bouzidi

**Affiliations:** a University of Tunis El Manar, Tunis Faculty of Medicine, 1007, Tunisia; b Pathology Department, Hospital Mongi Slim La Marsa, Tunisia; c Department of Orthopedic Surgery, Hospital Mongi Slim La Marsa, Tunisia; d Mohamed Kassab Institute of Orthopedics, Tunis, Tunisia

**Keywords:** Elbow, Infection, Tuberculosis, Osteo-arthritis pathology

## Abstract

**Introduction and importance:**

Tuberculous osteoarthritis, a rare condition affecting the elbow in 1–5 % of cases, poses diagnostic challenges due to its subtle clinical presentation, often resulting in delayed diagnosis. Herein, we present a case of tuberculous osteoarthritis involving the elbow joint. Our aim is to underscore the complexities associated with diagnosing this condition and to emphasize the critical importance of early recognition and appropriate management strategies for optimal patient outcomes.

**Case presentation:**

A 44-year-old Tunisian woman presented with a year-long history of painful right elbow swelling, systemic symptoms, and purulent drainage. Physical examination revealed a swollen, erythematous elbow with limited mobility. Radiographs showed periarticular osteolysis and subluxation, and *Mycobacterium tuberculosis* was cultured from the purulent drainage. The patient underwent open arthrotomy, synovectomy, joint irrigation, external fixation, and immobilization. Histopathology confirmed tuberculosis. She started a 12-month anti-tuberculous treatment and rehabilitation plan but was lost to follow-up due to socioeconomic difficulties.

**Clinical discussion:**

This case underscores the intricate diagnostic challenges of tuberculous osteoarthritis, emphasizing the necessity of a comprehensive assessment for precision in diagnosis. Timely intervention plays a pivotal role in averting joint deterioration and securing favorable results, especially in regions with high endemicity.

**Conclusions:**

Early recognition and management of tuberculous osteoarthritis are vital for preserving joint function. Maintaining a high suspicion for tuberculosis in cases of unusual joint symptoms is key to timely diagnosis and effective treatment, leading to improved patient care and outcomes.

## Highlights

• Tuberculous osteoarthritis should be considered in patients with mono/oligoarticular arthritis, notably in endemic areas.
• Elbow tuberculosis is diagnosed by positive *Mycobacterium tuberculosis* cultures and caseating granulomas on biopsies.
• *Mycobacterium tuberculosis* infection is confirmed through microbiological and histopathological tests.
• Differential diagnoses for elbow skeletal tuberculosis include pyogenic arthritis, pigmented villonodular synovitis, hemophilic arthropathy, rheumatoid arthritis, osteochondromatosis, tumors, sarcoid arthritis, and gout.
• Early detection is paramount to prevent irreversible joint damage and maintain functional capacity.
• Swift initiation of anti-tubercular therapy is critical for preserving joint integrity and function.
• Managing complex osteoarticular tuberculosis involves a multidisciplinary team of specialists

## 1 Introduction

Musculoskeletal tuberculosis is an uncommon condition, representing 35 % of extrapulmonary tuberculosis cases and comprising only 1–3 % of all tuberculosis cases worldwide [[1], [2], [3]]. The most commonly infected joints are the spine, hip, and knee in order of frequency. Elbow joint infection is particularly rare, making up 1–5 % of all cases of musculoskeletal tuberculosis [[1], [2], [3]]. The subtle and varied clinical features of elbow tuberculous osteoarthritis can lead to missed diagnoses. There is a need to better understand the optimal diagnostic and treatment approaches for this rare condition. Early detection is crucial to prevent irreversible joint damage and preserve functional capacity. Herein, we present a case of elbow tuberculous osteoarthritis, aiming to shed light on the diagnostic challenges and stress the importance of early recognition for improved patient outcomes.

This case report adheres to the SCARE Criteria [4].

## 2 Case presentation

A 44-year-old Tunisian woman with no significant medical history, including diabetes or immunosuppression, presented with progressive right elbow pain, swelling, and functional decline over the past year, which she had neglected. She also reported systemic symptoms such as fatigue, night sweats, low-grade fever, and general deterioration. From a rural area with unfavorable socioeconomic conditions, she had no history of trauma, septic inoculation, or tuberculosis exposure. Six months later, a sinus tract developed with purulent drainage, prompting her to seek emergency care. On examination, the elbow was swollen (10 cm), painful, erythematous, warm, and had limited mobility (30/80 range of motion), with no involvement of other joints. Standard radiological assessment, including anteroposterior and lateral plain radiographs of the right elbow, showed marginal periarticular osteolysis and elbow subluxation (Fig. 1A, Fig. 1B). Laboratory results revealed a C-Reactive Protein level of 40 mg/L (normal: 0–5 mg/L), Erythrocyte Sedimentation Rate of 37 mm/h (normal: 0–20 mm/h), and a white blood cell count of 11 × 10^9^/L (normal: 4.5–11 × 10^9^/L). The pus sample was cultured for bacteria, including acid-fast bacilli (AFB), and *Mycobacterium tuberculosis* was identified, confirming the diagnosis of tuberculosis. The preoperative chest radiograph was normal. Given the diagnosis of elbow osteoarthritis and the urgent situation, surgery was performed without further radiological investigation. An open arthrotomy was performed with a lateral approach to access the joint, excising the fistula and navigating between the anconeus and ulnar extensor muscles. During surgery, osteocartilaginous and capsuloligamentous destruction in the anterolateral region was noted, extending into the surrounding soft tissues. Hypertrophic synovium resembling a pseudotumor, bone erosion, and minimal purulent drainage were also observed (Fig. 2). The joint was then irrigated, drained, and underwent synovectomy. An external fixator was applied to stabilize the joint and address anterior-lateral instability, followed by joint immobilization (Fig. 3A, Fig. 3B). Histopathological examination of the synovial biopsy specimen revealed granulomatous inflammation with areas of caseous necrosis, surrounded by epithelioid histocytes, Langhans-type giant cells, and lymphocytes (Fig. 4A, Fig. 4B, Fig. 4C, Fig. 4D). The postoperative course was uneventful. The patient was presented to the multidisciplinary team for complex osteoarticular infections, and the decision was made to initiate anti-tuberculous treatment: a quadruple therapy consisting of rifampicin, isoniazid, ethambutol, and pyrazinamide for 2 months, followed by 10 months of dual therapy with rifampicin and isoniazid. The external fixator was kept in place for 15 days and then replaced with a hinged elbow brace. Given the patient's young age, a possible elbow arthroplasty was considered after performing control imaging and biological assessments. A postoperative rehabilitation plan was prescribed for this patient. Due to her unfavorable socioeconomic conditions, the patient was lost to follow-up shortly after starting treatment, removal of the external fixator, and wound healing. As a result, we were unable to monitor her clinical and radiological progress, assess side effects, or continue the planned treatment.

**Fig. 1A:**
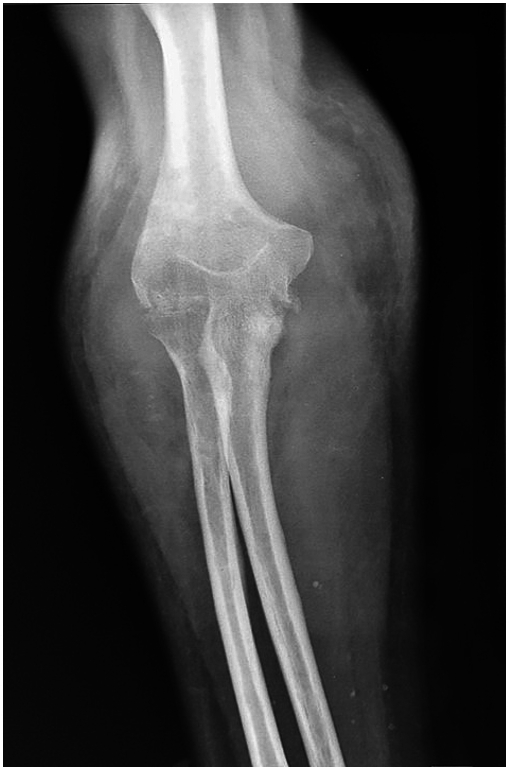
Anteroposterior radiograph of the right elbow showing marginal periarticular osteolysis and elbow subluxation.

**Fig. 1B:**
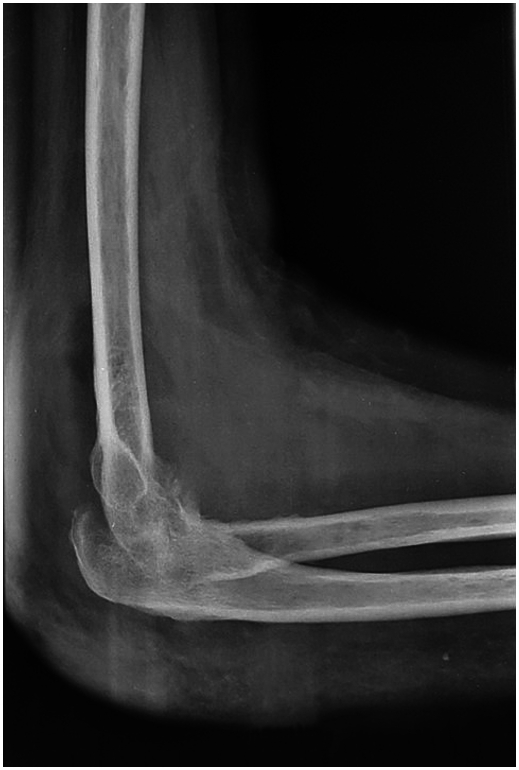
Lateral plain radiograph of the right elbow demonstrating marginal periarticular osteolysis and elbow subluxation.

**Fig. 2:**
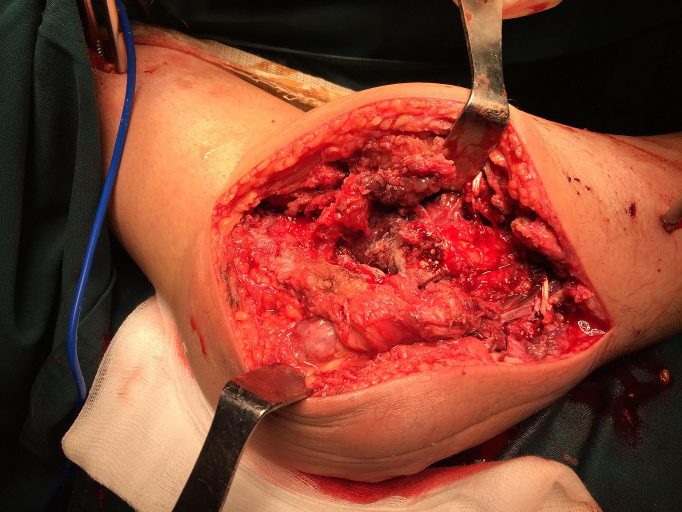
Intraoperative image showing the surgical exploration of the lateral aspect of the elbow. The image reveals osteocartilaginous and capsuloligamentous destruction in the anterolateral region, extending into the adjacent soft tissues. Additionally, there is hypertrophic synovium resembling a pseudotumor, accompanied by bone lysis and minimal purulent discharge.

**Fig. 3A:**
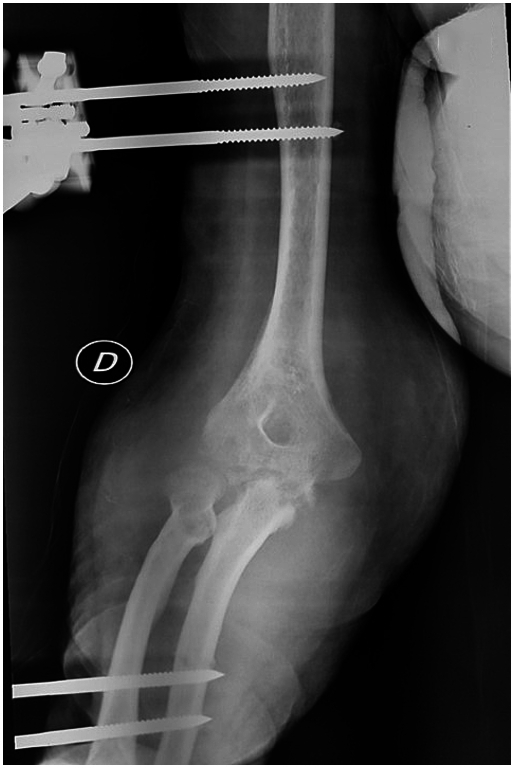
Anteroposterior radiograph of the right elbow demonstrating the utilization of an external fixator for stabilization.

**Fig. 3B:**
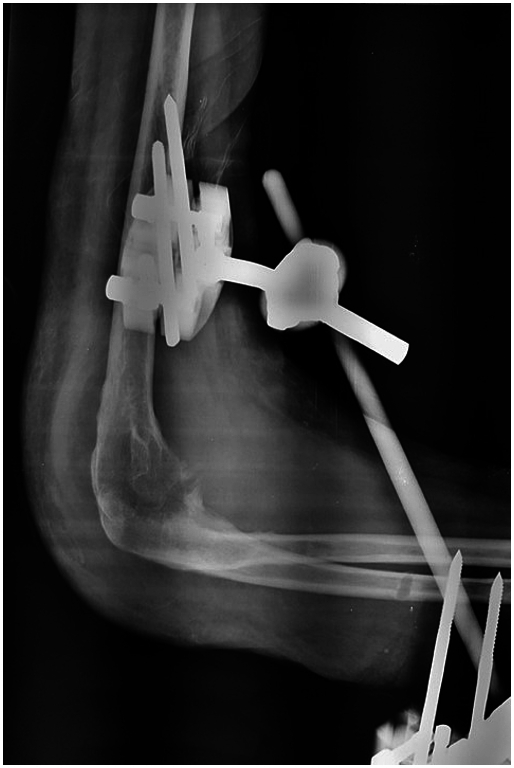
Lateral radiograph of the right elbow demonstrating successful stabilization achieved with an external fixator.

**Fig. 4A:**
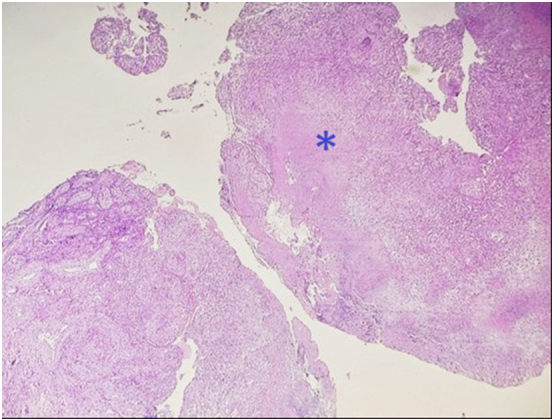
Histological examination showing granulomatous inflammation with caseous necrosis (asterisk), epithelioid cells, Langhans giant cells, and lymphocytic infiltration. (Hematoxylin and eosin, magnification × 40).

**Fig. 4B:**
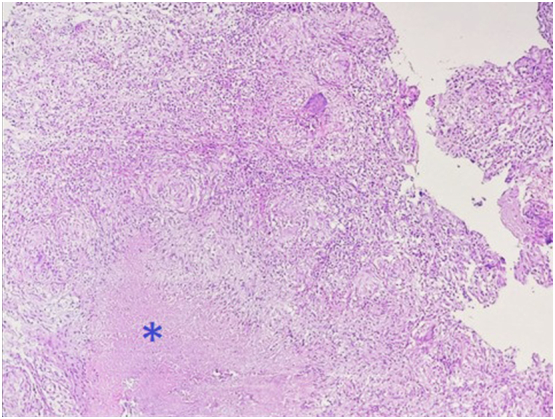
The image depicts the presence of caseating granulomas, characterized by central caseous necrosis (asterisk) surrounded by epithelioid cells, Langhans giant cells, and a dense infiltration of lymphocytes. (Hematoxylin and eosin, magnification × 100).

**Fig. 4C:**
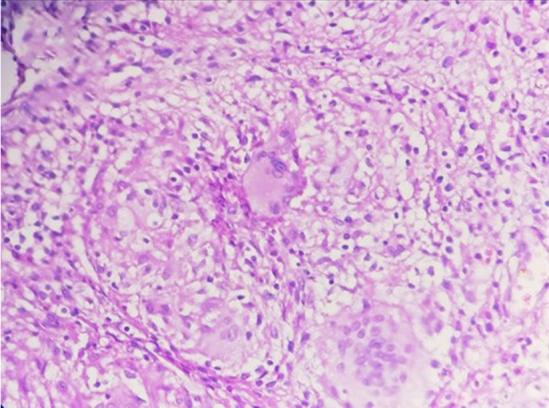
Epithelioid granuloma with multinucleated Langhans giant cells (Hematoxylin and eosin, magnification × 400).

**Fig. 4D:**
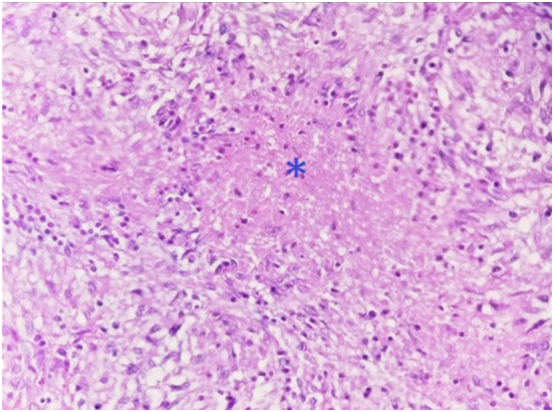
Caseous necrosis (asterisk) surrounded by epithelioid cells (Hematoxylin and eosin, magnification × 400).

## 3 Discussion

In 2019, extrapulmonary tuberculosis accounted for 16 % of the 7.5 million global cases, ranging from 8 % in the WHO Western Pacific region to 24 % in the Eastern Mediterranean region [5]. Certain groups, such as younger individuals, females, and those of African or Asian descent, are at higher risk. Musculoskeletal tuberculosis comprises 10 % to 25 % of extrapulmonary cases, with an estimated global prevalence of 19 to 38 million [5,6]. The spine is most commonly affected (50 % to 69 %), followed by the hip, knee, and ankle/ft (10 % to 13 %) [5,6]. Tuberculous osteoarthritis of the elbow is rare, representing 1–5 % of musculoskeletal tuberculosis cases. It typically arises from a latent pulmonary focus or another extrapulmonary infection site and spreads via hematogenous dissemination, lymphatic spread, or direct bacilli transmission [7]. Elbow tuberculosis typically begins at the olecranon or distal humerus, although it can also affect the synovium or proximal radius in some cases [8]. The lesion combines osteomyelitis and arthritis, with initial synovial inflammation leading to granulation tissue formation, effusion, and fibrin deposition, which may result in “rice” bodies [8]. This granulation tissue pannus then proceeds to erode cartilage, causing bone demineralization and caseous necrosis [8,9]. Tuberculous osteoarthritis typically presents insidiously with chronic joint pain, mild swelling, and gradual functional loss. The pain worsens with movement, while swelling is less pronounced than in other types of arthritis. As the condition progresses, stiffness and reduced range of motion impair daily activities. Constitutional symptoms, including low-grade fever, weight loss, night sweats, and anemia, occur in some cases, contributing to the gradual onset of the disease [8,9]. In approximately 50 % of the cases, symptoms and radiographic evidence of pulmonary tuberculosis are absent, and tuberculosis is frequently missed as a differential diagnosis of the chronic inflammation of joints in the absence of active pulmonary disease [10]. In our case, the preoperative chest radiograph revealed normal findings. A tuberculin skin test, typically positive in musculoskeletal tuberculosis cases, stands as a valuable diagnostic tool. Verification through microscopy and culture remains essential, with joint fluid aspirate evaluation for smear and culture often yielding positive results in 80 % of instances [7,8]. A clear synovial aspirate serves as an excellent specimen for polymerase chain reaction and nucleic acid probes. On plain radiographs, the Phemister triad, indicating tuberculosis arthritis, may be visible, comprising juxta-articular osteopenia, peripherally situated osseous erosions, and a gradual joint space narrowing [8,11,12]. Martini et al. classified the radiological display of osteoarticular tuberculosis into four stages: localized osteoporosis without bony lesions (stage 1), one or more erosions or cavities in bones (stage 2), joint involvement without significant destruction (stage 3), and gross destruction (stage 4) [11,12]. CT scans are useful for assessing bone degradation, soft tissue involvement, and sequestrum formation [12,13]. MRI effectively visualizes signs of osteomyelitis, including bone marrow edema, chondral and subchondral bone erosion, synovial thickening, joint effusion, joint space narrowing, and soft tissue spread. Bone marrow edema appears hypointense on T1-weighted and hyperintense on T2-weighted images with gadolinium enhancement [13,14]. In tuberculosis arthritis, synovial thickening appears hypointense on T2-weighted images, distinguishing it from other synovial disorders. While imaging provides important insights into tuberculosis involvement in the elbow, a definitive diagnosis requires biopsy specimens from affected tissue, synovium, or bone, with positive *Mycobacterium tuberculosis* cultures and caseating granulomas. The differential diagnosis of elbow tuberculous osteoarthritis includes pyogenic arthritis, which presents acutely with fever and purulent effusion, and rheumatoid arthritis, which causes chronic inflammation and deformity, distinguishable by autoantibodies. Osteoarthritis, due to mechanical wear, shows joint space narrowing, osteophytes, and subchondral sclerosis but lacks infection signs. Conditions like brucellosis, fungal infections, gout, and pseudogout can mimic tuberculosis but are distinguishable by risk factors or crystal analysis. Trauma and avascular necrosis cause elbow pain, with specific radiographic features, such as bone collapse in necrosis, unlike tuberculosis [7,8,11]. The primary treatment approach for musculoskeletal Tuberculosis involves three to four drug regimens based on sensitivity findings. While the optimal treatment duration remains uncertain, conventional therapy spans 12–18 months [7,8]. Recent evidence suggests that regimens containing rifampin can be effective with a treatment duration of six to nine months [15]. Timely diagnosis and anti-tuberculous chemotherapy coupled with early mobilization can facilitate significant functional recovery. Surgery is seldom required in the contemporary era due to the advent of chemotherapy. Advanced cases of peripheral joint involvement can be effectively managed with synovectomy, necrotic tissue debridement, and abscess drainage, obviating the need for arthrodesis. Surgery should be considered only in cases where deformities persist or function improvement is required if the disease does not respond adequately to chemotherapy [7,8].

In conclusion, tuberculous osteoarthritis of the elbow, though rare, presents with subtle symptoms that can easily mimic other joint disorders, making early diagnosis challenging. Delayed diagnosis can lead to severe, irreversible consequences, including joint destruction, ankylosis, and significant functional impairment. Without timely intervention, the infection progressively damages cartilage, bone, and soft tissues, resulting in permanent deformities. Physicians must maintain a high index of suspicion, particularly in regions where tuberculosis is endemic, such as Tunisia. A comprehensive and effective management of complex cases of osteoarticular tuberculosis necessitates a multidisciplinary approach, involving orthopedic surgery, microbiology, and infectious disease specialists to ensure optimal care and treatment. Swift initiation of anti-tubercular therapy is essential to preserve joint integrity and function.

## Consent

Written informed consent was obtained from the patient for publication of this case report and accompanying images. A copy of the written consent is available for review by the Editor-in-Chief of this journal on request.

## Provenance and peer review

Not commissioned, externally peer-reviewed.

## Ethical approval

Ethical approval for this study was provided by the Ethical Committee of Mongi Slim University Hospital, Marsa, Tunisia.

## Sources of funding

This research did not receive any specific grant from funding agencies in the public, commercial, or not-for-profit sectors.

## Author contribution

**Dr. Faten LIMAIEM** and **Dr Leila BOUHAJJA:** Prepared, organized, wrote, and edited all aspects of the manuscript.

**Dr. Mohamed Amine GHARBI,** and **Pr. Ramzi BOUZIDI:** Read, edited, and approved the final version of the manuscript. Contributed to data acquisition, analysis, and interpretation. Provided final approval of the manuscript before its submission.

## Guarantor

Dr. Faten LIMAIEM.

## Research registration number

N/A

## Conflict of interest statement

None declared.
